# Signal to noise ratio quantifies the contribution of spectral channels to classification of human head and neck tissues *ex vivo* using deep learning and multispectral imaging

**DOI:** 10.1117/1.JBO.28.1.016004

**Published:** 2023-01-28

**Authors:** George S. Liu, Jared A. Shenson, Joyce E. Farrell, Nikolas H. Blevins

**Affiliations:** a Stanford University, Department of Otolaryngology — Head and Neck Surgery, Palo Alto, California, United States; b Stanford University, Department of Electrical Engineering, Stanford, California, United States

**Keywords:** multispectral imaging, machine learning, classification, lasers, sensor, tissue

## Abstract

**Significance:**

Accurate identification of tissues is critical for performing safe surgery. Combining multispectral imaging (MSI) with deep learning is a promising approach to increasing tissue discrimination and classification. Evaluating the contributions of spectral channels to tissue discrimination is important for improving MSI systems.

**Aim:**

Develop a metric to quantify the contributions of individual spectral channels to tissue classification in MSI.

**Approach:**

MSI was integrated into a digital operating microscope with three sensors and seven illuminants. Two convolutional neural network (CNN) models were trained to classify 11 head and neck tissue types using white light (RGB) or MSI images. The signal to noise ratio (SNR) of spectral channels was compared with the impact of channels on tissue classification performance as determined using CNN visualization methods.

**Results:**

Overall tissue classification accuracy was higher with use of MSI images compared with RGB images, both for classification of all 11 tissue types and binary classification of nerve and parotid (p<0.001). Removing spectral channels with SNR>20 reduced tissue classification accuracy.

**Conclusions:**

The spectral channel SNR is a useful metric for both understanding CNN tissue classification and quantifying the contributions of different spectral channels in an MSI system.

## 1 Introduction

Precise identification of tissues based on their visual appearance is a challenge in surgery. Tissues can be difficult to distinguish based on their appearance under conventional white light illumination, highlighting the need for additional sources of information, such as tactile feedback, intraoperative nerve monitoring devices, and image-guided navigation software. The widespread use of operative microscopes and endoscopes has made it possible to augment the surgeon’s vision with information obtained from new imaging technologies, such as narrowband,^1^ multispectral,^2^ and hyperspectral^3^ imaging.

Hyperspectral imaging (HSI) captures many different narrowband spectral images of the same scene or specimen. The complete set of images spans a wide range of wavelengths and is represented as a hyperspectral image datacube. HSI is used in many important scientific and industrial applications. However, the time and expense that is necessary to capture a full hyperspectral image datacube make it impractical for many surgical applications. Multispectral imaging (MSI) captures images with fewer spectral bands than HSI but with more than the three (RGB) spectral bands that are available in most digital cameras. The reduction in time and expense for data capture makes MSI feasible for surgical applications.

There are many review papers summarizing the progress that has been made in HSI,^3^^–^^10^ and many papers exploring the use of HSI for tissue classification. For example, HSI has been used to classify cancerous and normal tissue in the colon,^11^^,^^12^ prostate,^13^ head and neck tissues,^14^^–^^16^ oral cavity,^17^ liver,^18^ breast,^19^^,^^20^ and brain.^21^ The vast majority of these studies use linear classifiers, such as support vector machines (SVM), which are ideal for binary classification tasks, such as discriminating healthy from unhealthy tissue or benign from malignant tumors. Linear classifiers trained on HSI systems can also lead to insights about the features that are important for differentiating tissues.^22^^,^^23^ More recently deep learning approaches have been used to differentiate normal and pathologic tissues with applications to identifying cancer margins including in the head and neck.^16^^,^^24^

Advances in machine learning have made it possible to train deep convolutional networks (CNNs) to perform multiclass classification tasks, such as labeling the type or grade of cancerous tissue or the anatomical origin of the tissue. Although multiclass classification by CNNs can outperform classification by human experts,^2^ they remain a blackbox that does not provide insights into how they perform and consequently how they can be improved.

This paper describes an in-depth analysis of the performance of a CNN that was trained to classify different head and neck tissues that were captured by an MSI system *ex vivo*. In a previous paper, we reported that MSI imaging improved the accuracy of tissue classification over RGB imaging, and a CNN trained on MSI image data produced fewer tissue classification errors than surgical residents in otolaryngology.^2^ In this paper, we use data visualization methods, such as occlusion analysis^25^ and tissue confusion matrices, to explain the performance and decision-making process of the CNN trained to classify the different tissue types. Our analysis reveals that the signal-to-noise ratio (SNR) is a useful metric to quantify the impact that each spectral channel in the MSI system has on tissue classification.

## 2 Methods

### 2.1 Multispectral Imaging System Design and Calibration

**Fig. 1:**
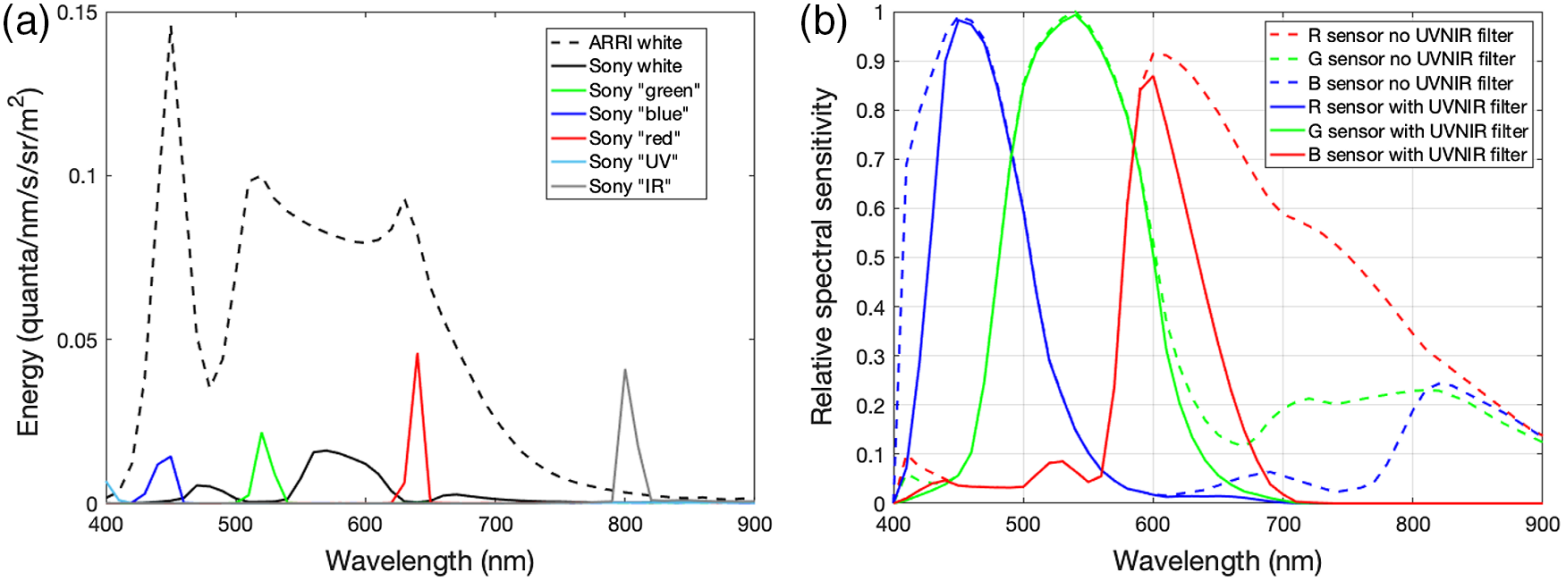
(a) Spectral energy in seven different light sources. “ARRI white” refers to the broadband LED light in the ARRIscope. All other lights were created by the external Sony laser light source. (b) Estimated spectral sensitivities of the RGB sensors in the ARRIScope with (solid lines) and without (dashed lines) a UV + NIR blocking filter. See Supplemental Material for a description of the method used to derive these functions.

An MSI system was created by combining a fully digital stereoscopic operating microscope (the ARRIScope, manufactured by Munich Surgical Medical), with an external laser light source (RGBW-G5; Sony Corp) and a fiber-optic ring-light illuminator (Boli Optics). Sequential illumination was performed using broadband white light (400 to 810 nm) from the ARRIScope’s native LED white light source, white light (450 to 700 nm) from the external Sony laser light source, and narrowband blue (445 nm), green (525 nm), red (638 nm), ultraviolet (UV) (405 nm), and infrared (IR) (808 nm) lights, also from the Sony light source, creating a total of 7 illumination conditions. The radiant power for all the lights is less than the MPE for all the hazard conditions defined in IEC 62471:2006 and ANSI X136.1:201. Figure 1(a) plots the spectral energy in each of the light sources, measured by a PR-715 SpectraScan spectroradiometer (Photo Research). The combination of the seven lights and the three RGB imaging sensors in the ARRIScope created an MSI system with 21 spectral channels.

We calculated the signal-to-noise ratio for each of the 21 spectral channels based on the following assumptions. First, we assume that the maximum signal that a spectral channel can measure is determined by the spectral energy of the light that is incident on the sensor, the spectral quantum efficiency of the sensor, the sensor pixel size, exposure duration, and conversion gain. We measured the spectral energy of the lights [shown in Fig. 1(a)] and estimated the spectral quantum efficiency of the RGB sensors [shown in Fig. 1(b)]. The method we used to estimate the spectral quantum efficiency of the RGB sensors is described in the Supplemental Material.

Second, we define the signal-to-noise ratio of each spectral channel as the ratio of the maximum signal (in this case, calculated in units of photons) divided by the sensor noise. Even a sensor that has no intrinsic electronic noise will generate a signal (electrons) that includes noise (random fluctuations) due to the inherent variation of incident photons. Photon noise can be characterized by a Poisson distribution such that the noise is equal to the square root of the number of photons.

Our calculation of spectral channel SNR is based on the maximum number of photons that a spectral channel can measure in a fixed amount of time divided by photon noise. We do not take into account sensor noise, although it is worth noting that current CMOS imaging sensors have very little sensor noise, which is typically less than two electrons. Our calculation of the signal is based on the maximum signal that each spectral channel can measure. Hence, in this case, spectral channel SNR is also a measure of the spectral channel dynamic range.

We use the spectral channel SNR to quantify the capacity of each channel to capture spectral energy. The actual signal measured by each spectral channel will be the product of the spectral energy of the light, the spectral quantum efficiency of the sensor, and the percentage of the light that is reflected and scattered by the tissue. The signal may also include light that is emitted by the tissue due to autofluorescence. Because we do not customize our illumination light source and do not block reflected light from the sensor, tissue autofluorescence, if present, will be weak compared with the diffuse tissue reflectance detected by the RGB sensors and is unlikely to be differentiated from sensor noise.

The exposure duration and conversion gain were held constant for our measurements. Hence, the maximum number of photons for each channel can be calculated by converting the product of the spectral energy of the light and the estimated sensor spectral sensitivity into units of photons, and then taking the sum across all wavelengths. For the purpose of this calculation, we assume a 1  μm pixel and a 30 millisecond (ms) exposure and convert ${\text{photons}/\;m}^{2}/s$ to $\text{photons}/\mu m^{2}/30\;\mathrm{ms}$. Then, we calculate the SNR in decibels by $20*\log\;10(S/S^{0.5})$ where S is the number of photons captured by a 1  μm pixel in a 30 ms exposure.

Table 1 lists the SNR for the 21 spectral channels created by combining the estimated spectral sensitivities of the RGB sensors in the ARRIScope with the spectra power of the 7 different lights. With the exception of the IR (808 nm) illumination condition, images were captured with an NIR blocking filter placed in front of the ARRIScope RGB sensors. The estimated spectral sensitivities of the RGB sensors (see Supplemental Material) predict that the sensor would not be able to detect the UV (405 nm) light. This explains why the SNR for the spectral channels corresponding to the R, G, and B sensors with the UV (405 nm) light has low SNR (8.75, 12.3, and 13.7, respectively). The estimated spectral sensitivities of the RGB sensors predict that the sensor can measure the spectral energy in the 808 nm light. This explains why the SNR for the three spectral channels corresponding to the R, G, and B sensors combined with the IR illumination (808 nm) has relatively high SNR when the NIR blocking filter is removed (34.2, 32.6, 32.1, respectively). The spectral channels with the highest SNR are the three spectral channels corresponding to the R, G, and B sensors with the ARRIScope broadband light (46.67, 49, 46.6, respectively). The SNR for the three spectral channels created by combining the R, G, and B sensors with the Sony broadband light was also relatively high (36.3, 38.1, 31.8, respectively). Finally, as one would expect, the SNR of spectral channels created by combining R sensors with a “red” (636 nm) light, G sensors with a “green” (525 nm) light, and B sensors with a “blue” (445 nm) light, are also high (33.5, 34.0, 32.5).

**Table 1:** Spectral channel SNR.

	ARRI broadband broad white	Sony broadband narrow white	Sony 405 nm UV	Sony 445 nm blue	Sony 525 nm green	Sony 638 nm red	Sony 808 nm IR
R	46.44	36.4	8.76	20.15	23.93	33.55	34.49
G	49.01	38.20	12.33	21.08	34.06	26.95	32.67
B	46.61	31.86	13.71	32.56	28.75	19.21	32.14

### 2.2 Data Acquisition

A surgically trained investigator (JAS) dissected 92 head and neck tissue specimens from 9 fresh-preserved human cadavers. The investigator used anatomical information to classify the tissue specimens into one of 11 tissue types: artery, bone, cartilage, dura, fascia, fat, muscle, nerve, parotid, skin, and vein. Additionally, two images of nerve and parotid that were overlapping or adjacent *in situ* were obtained.

The tissue specimens were imaged using a black box to contain the specimen, a blackout drape around the imaging system, and by turning off all room lighting to reduce ambient light during imaging.

MSIs were captured by sequentially illuminating each tissue sample with the seven lights to create the MSI dataset containing 21 spectral bands (three sensors and seven spectral lights). Non-multispectral images captured with the ARRIScope LED white light only will be referred to as the RGB dataset, to distinguish this dataset (three sensors and one spectral light) from the MSI dataset.

Images acquired on the ARRIScope were saved as ARRIRAW unprocessed sensor data and TIFF processed RGB images. Both formats contained images with spatial dimensions of 1920×1080  pixels. The ARRIRAW data were used to develop and test CNN models. TIFF images were used to visualize images and create manually segmented binary masks of tissue foregrounds. See Supplemental Material for example TIFF images obtained by the MSI system for each of the 11 tissue types.

### 2.3 Dataset

The ARRIScope saved ARRIRAW sensor data with binocular images from the left and right microscope eyepieces. However, corresponding TIFF images contained monocular images from the left eyepiece only. Therefore, only left eyepiece images from ARRIRAW data were used in data analysis. ARRIRAW sensor data were processed in MATLAB (The Mathworks) using publicly available MATLAB code^26^ to demosaic, rotate, and isolate the left eyepiece images and match the orientation of corresponding TIFF images. After processing, ARRIRAW images were saved as MATLAB data files (MAT-files) for use in Python. All further image analysis was performed using Python 3.7.

**Fig. 2:**
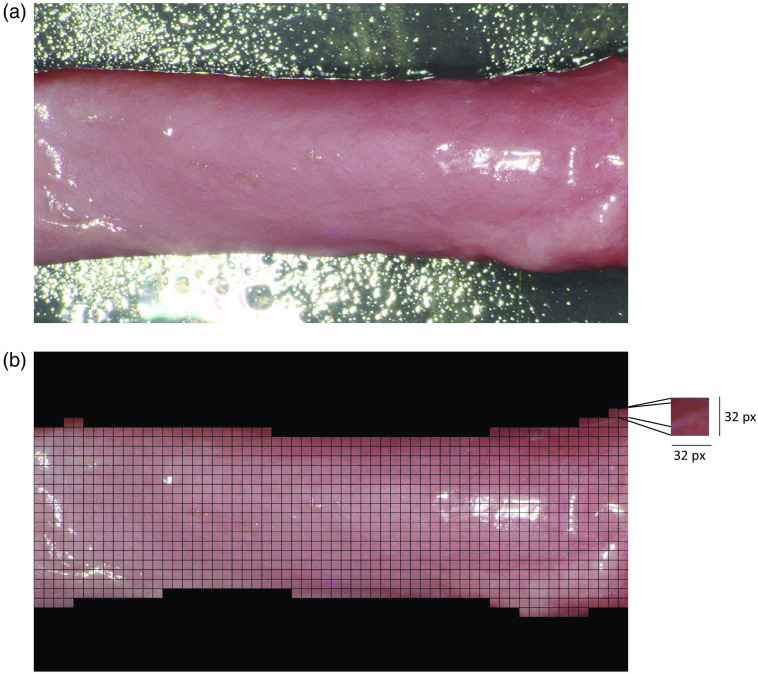
Image processing steps to convert an original-sized image into image patches. (a) Original-sized image of artery (from the test dataset) with spatial dimensions of 1920×1080  pixels. (b) The original-sized image is divided into contiguous, non-overlapping image patches of size 32×32  pixels. Image patches containing any pixel of background, as determined from a hand-segmentation of tissue foreground, are omitted and colored black. Here, there are 832 image patches of artery foreground. The top-right-most image patch is enlarged to show detail. The specularity in the broad-band white illuminated image shown in this figure is an artifact of how the images are processed and rendered by the ARRIScope imaging system. Raw unprocessed image data were used to train the deep-learning neural networks. Raw pixel values that appeared as white in the rendered TIFF images were analyzed and it was found that >99% of the pixels were not saturated (data not shown).

Manual segmentation was performed by two investigators with surgical training (GSL and JAS) using anatomical information of tissue to identify ground truth segmentations. Segmentations were performed on TIFF images using the LabelBox online platform.^27^ The resulting binary masks were used to identify tissue foreground in corresponding ARRIRAW images. These regions of tissue foreground were tiled into 32×32  pixels patches that were input to CNN models (see Fig. 2). Image patches containing only tissue foreground were used; image patches containing any background pixels were omitted to avoid the use of background noise, which would degrade model performance.

A total of 56,314, 22,836, and 12,383 image patches were present in the train, validation, and test datasets, as shown in Table 2 and described previously.^2^ Of note, the datasets contain class imbalance with an uneven representation of tissue types. Under-represented tissue types in the combined dataset include nerve (2356 image patches), bone (5764 image patches), and cartilage (6006 image patches), whereas over-represented tissue types include muscle (11,830 image patches), skin (11,467 image patches), and fascia (11,018 image patches).

**Table 2:** Number of cadaver specimens and image patches (32×32  pixels) in the training, validation, and test data sets for each tissue type.

	Training		Validation		Test	
Tissue	Cadavers	Patches	Cadavers	Patches	Cadavers	Patches
Artery	5	4411	2	1722	1	832
Bone	5	3446	2	1668	1	650
Cartilage	5	3940	2	1611	1	455
Dura	5	4632	2	2260	1	1394
Fascia	6	6815	2	2846	1	1357
Fat	6	7017	2	2464	1	1513
Muscle	6	7529	2	2774	1	1527
Nerve	6	1631	2	354	1	371
Parotid	5	4661	2	2407	1	1534
Skin	5	7212	2	2721	1	1534
Vein	5	5020	2	2009	1	1216
Total		56,314		22,836		12,383

#### 2.3.1 Architecture of CNN models

The RGB and MSI datasets contained 32×32  pixel image patches obtained from full-sized (1920×1080  pixels) images of tissues from fresh preserved human cadavers.^2^ Both datasets were partitioned into train, validation, and test sets using tissues from six, two, and one cadavers, i.e., there were six cadavers in the training set, two cadavers in the validation set, and one cadaver in the test set. Care was taken to ensure that the train, validation, and test sets contained tissue specimens from different cadavers. The train and validation sets were used to develop candidate CNN models and select the best-trained model for testing using criteria of overall validation accuracy. The test set was used for final evaluation of the best-trained CNN model on held out data. A separate hold out set of nerve and parotid *in situ* images were used for a secondary evaluation of the best-trained models on images with more than one tissue type present.

We developed two CNN models, called ARRInet-W and ARRInet-M, that were trained using a one-vs-all approach to classify the 11 different tissue types in the RGB and MSI datasets, respectively. Both models used the Densenet CNN architecture,^28^ which applies a dense connectivity pattern between feature maps to reduce the total number of model parameters and achieve high performance in image classification tasks.^29^^,^^30^ Again, the inputs to ARRInet-W and ARRInet-M were image patches with spatial dimensions of 32×32  pixels and spectral dimension of either three channels for inputs to ARRInet-W (three RGB channels for broad white light illumination only) or 18 channels for inputs to ARRInet-M (three RGB channels for each of the six illumination conditions). The model outputs were 11-element vectors containing the predicted probabilities for the 11 tissue classes. The tissue class with the highest predicted probability was determined to be the final tissue prediction.

### 2.4 Development of CNN Models

CNN models were trained from scratch using the PyTorch framework^31^ in Python 3.7. Training was performed using a method similar to that described in Shenson et al.^2^ with the exception that ARRIRAW data were used as image inputs rather than processed TIFF images. More specifically, the ARRIRAW data were demosaicked and rotated, but no additional image transformations were applied. The reason for this was to avoid data distortions introduced by unknown (proprietary) image transformations due to color balancing and possible non-linear image enhancements. CNN models were trained using stochastic gradient descent with a multiclass cross-entropy loss function for backpropagation. The learning rate was initialized to 0.001 and updated with Adam, a stochastic gradient-based optimizer with adaptive learning rates.^32^ L2 regularization with a value of 0.001 was used to reduce overfitting. Hyperparameters were determined by an ad hoc search to maximize the classification accuracy of trained models on the validation dataset. The network was trained for 40 epochs in batches of 128 images. Deep learning computations were performed on an NVIDIA GeForce RTX 2070 8 GB GPU graphics card configured with NVIDIA CUDA on an MSI Trident X 9SD-021US desktop computer, using the PyTorch framework.^33^

#### 2.4.1 Evaluation of CNN models

Because the train set contained substantial class imbalance (Table 2), we used the total accuracy irrespective of class (microaverage accuracy) as the criteria^34^ for selecting the best-trained candidate models. The best-trained CNN models, ARRInet-W and ARRInet-M, were evaluated on image patches in the hold-out test set to quantify classification accuracy when applied to previously unseen images. Test performance metrics included sensitivity and specificity of one-vs-all multiclass classification, the average probability of correct tissue predictions, and micro-average accuracy. Receiver operating characteristic (ROC) curves for one-vs-all classification for each tissue type were generated using the scikit-learn toolbox in Python.^35^ In addition to the test set, two images of nerve and parotid tissues *in situ* were used to calculate the accuracies of the best-trained CNN models when applied to previously unseen images with overlapping or adjacent tissue configurations.

Secondary analysis was conducted using spectral occlusion sensitivity maps^25^ to assess the contributions of individual spectral channels to MSI image classification. Spectral occlusion sensitivity maps were generated by digital postprocessing of MSIs to simulate the effect of omitting one spectral channel (defined by an illumination-sensor combination) on MSI classification. This was done by replacing a single channel in an MSI image with the average value in that channel across the entire training dataset (across all tissue types). The absolute change in predicted probability of the correct tissue class indicates the contribution of the spectral channel to classifying the tissue type in the MSI image. Occlusion of spectral channels that are informative for classifying a tissue type cause a marked change in its probability score. To analyze large images, the average probability score across all foreground image patches was used before and after occlusion.

#### 2.4.2 Visualization of model performance

Prediction label maps and probability heat maps were generated to visualize areas of images that were correctly or incorrectly classified by the best-trained convolutional neural networks and provide insight into sources of prediction errors. Prediction label maps were generated for MSI and RGB image classification by moving a 32×32  pixels mask around the image and replacing the mask with a colored patch whose color indicated the predicted tissue type. Probability heat maps were generated by performing a similar procedure, except that the colored patch indicated the probability of a correct tissue prediction in the masked area.

### 2.5 Statistics

Chi-square statistics were computed in Microsoft Excel (Microsoft Corporation) to assess the significance of differences between tissue classification based on MSI and RGB imaging. DeLong’s method^36^ was implemented in Python to assess significance of differences between area under the curve (AUC) values of ROC curves. A critical alpha level of 0.001 was used to determine statistical significance.

## 3 Results

### 3.1 Classification Accuracy

The best-trained CNN models for RGB and MSI image classification, ARRInet-W and ARRInet-M, respectively, were evaluated on the hold-out test set. ARRInet-M demonstrated higher test classification accuracy for MSI images than did ARRInet-W for RGB images (Table 3). ARRInet-M and ARRInet-W demonstrated test macro average accuracies of 64.9% and 48.1%, respectively, and microaverage accuracies of 69.6% and 55.7%, respectively. Additionally, ARRInet-M predicted the correct tissue class with a higher probability, on average, compared with ARRInet-W, with macro average probabilities of 62.6% and 46.1%, respectively, and micro average probabilities of 67.0% and 53.0%, respectively. These accuracy metrics for ARRInet-M were all statistically higher than the accuracy metrics for ARRInet-W (p<0.001). Of note, test accuracy of tissue classification by ARRInet-M trained on processed (TIFF) and raw (ARRIRAW) image data was not significantly different (71.4% versus 69.6% microaverage accuracy, p=0.002).

**Table 3:** Multiclass classification performance results for multispectral and nonmultispectral classifiers, ARRInet-M and ARRInet-W, respectively, across all test image patches. Results are described as the percentage of correctly predicted tiles (“prediction”) and the average probability of a correct tissue prediction (“probability”).

	Multispectral imaging		RGB imaging	
	Prediction (%)	Probability (%)	Prediction (%)	Probability (%)
**Macroaverage**	**64.9**	**62.6**	**48.1**	**46.1**
**Microaverage**	**69.6**	**67.0**	**55.7**	**53.0**

The finding that classification accuracy was slightly better for networks trained on MSI images supports the assumption that increasing the number of spectral channels provides additional information that can be used to distinguish tissue types. In the next section, we quantify how important each spectral channel was in determining classification accuracy.

### 3.2 Occlusion Analysis

**Fig. 3:**
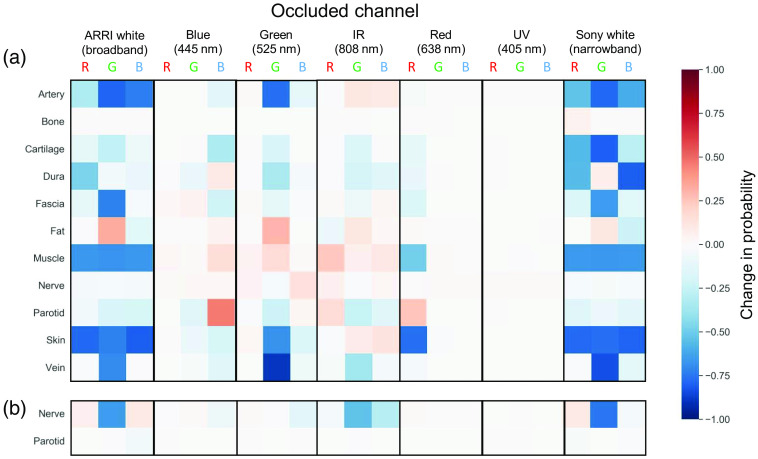
Spectral occlusion sensitivity maps displaying the effect of removing a single spectral channel on the overall probability of a correct tissue prediction by the multispectral classifier (ARRInet-M). Column labels indicate the illumination light source (and spectra) and RGB sensor (i.e., R, G, or B) of the occluded channel. Red and blue hues indicate increased and decreased tissue classification performance, respectively, with the indicated channel removed. (a) Occlusion maps for the 11-class multiclass classification task. Column labels at the top of the map designate channel spectra by the combination of illumination sequence (ARRIScope’s native LED broadband white light, external Sony laser blue light, etc.) and camera sensor (red, green, or blue) used for their acquisition. (b) Occlusion maps for the binary classification task of distinguishing nerve and parotid tissues only. Maps show results for test image classification.

**Fig. 4:**
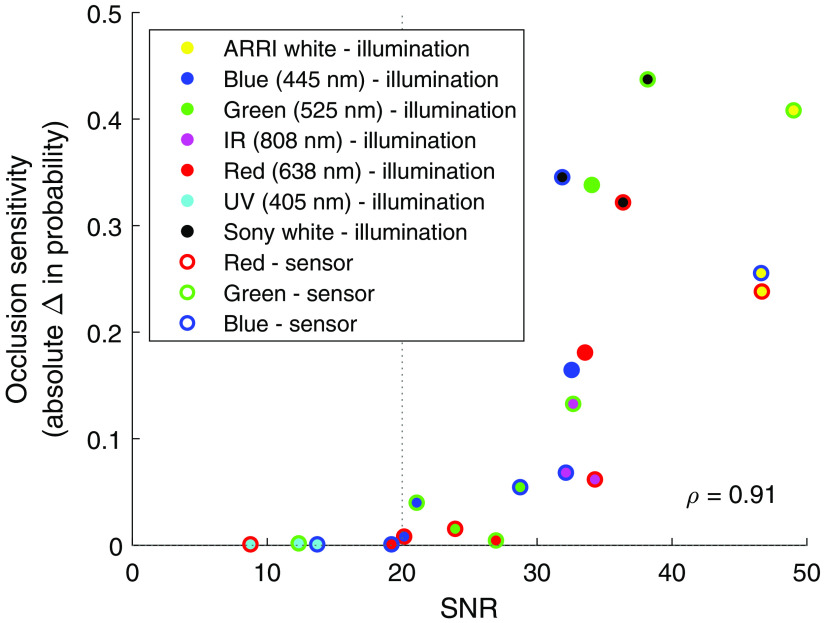
Scatter plot of each multispectral channel’s SNR and occlusion sensitivity in test image classification by ARRInet-M. Occlusion sensitivity is calculated as the absolute change in probability of correct tissue classification averaged across all tissue types after removing a spectral channel. A positive value indicates positive contribution of the channel to multispectral classification. Edge and face colors of points indicate the sensor and illumination spectra, respectively. Spearman’s rank correlation coefficient (ρ) between SNR and occlusion sensitivity is 0.91. Dotted vertical line indicates the SNR lower bound of 20 below which channels are noncontributory to classification.

We assessed the contribution of each spectral channel by removing it from the test data and observing the effect on the classification accuracy of ARRInet-M. Figure 3 shows occlusion sensitivity maps that illustrate how removing an individual spectral channel changes the accuracy of MSI classification by ARRInet-M. Spectral channels were ranked based on the absolute change in the probability of correct tissue classification on test images when the channel was occluded, averaged across all tissue types. This rank list was then compared with a second rank list calculated based on the SNR (Table 1). The Spearman rank-order correlation of spectral channels ranked by occlusion and by the channel SNR was 0.91, indicating a strong relationship between a channel’s contribution to classification and its SNR. A plot of SNR versus occlusion sensitivity (change in classification accuracy averaged across all tissue types) is shown in Fig. 4.

Removing spectral channels that have SNR<20 does not affect tissue classification errors. Removing spectral channels with SNR>20 produced large errors, with a few notable exceptions. Classification errors for bone remained large, regardless of whether spectral channels were removed or not. Removing spectral channels with high SNR improved classification in a few cases. Examples of these channels are the blue illumination-blue sensor combination for parotid tissue and ARRI white illumination-green sensor and green illumination-green sensor combinations for fat tissues (Fig. 3). Manual review of these spectral channels for parotid and fat image did not reveal distinguishing abnormalities of these images that would complicate their classification. A likely explanation is that the neural network model weights were overfit to these high SNR channels during training, and inaccurately interpreted them to classify the test image tissues. The size of our image dataset is small relative to the heterogeneity of possible tissue appearances in images, which increased the risk of overfitting with our dataset. The UV (405 nm) illumination channels had low SNR<20 and no contribution to occlusion analysis, likely because the illumination fell outside the spectral range of the RGB sensors in the ARRIScope.

### 3.3 Classification Errors

See Table 4 for a breakdown of test classification accuracies of ARRInet-W and ARRInet-M by tissue type. Both models exhibited a wide range of test accuracies, ranging from <1% for classifying bone to >90% for classifying fascia. Both models demonstrated good to excellent classification accuracy (>70%) for artery, fascia, muscle, and skin, and poor accuracy (<10%) for bone and nerve. Although ARRInet-M demonstrated much higher accuracy than ARRInet-W (>30% difference) for cartilage, dura, and vein, ARRInet-W demonstrated higher classification accuracy than ARRInet-M for bone, fat, muscle, parotid, and skin.

**Table 4:** Multiclass classification performance results for multispectral and nonmultispectral classifiers, ARRInet-M and ARRInet-W, for each tissue type. Results are described as the percentage of correctly predicted tiles (“prediction”) and the average probability of a correct tissue prediction (“probability”).

	Multispectral imaging		RGB imaging	
Tissue type	Prediction (%)	Probability (%)	Prediction (%)	Probability (%)
Artery	83.3	81.8	73	66.5
Bone	0	0.2	0.2	4.2
Cartilage	99.1	96.3	44.5	41.1
Dura	92.5	87.7	7.4	10.3
Fascia	93.4	87.9	94.2	91.4
Fat	51.7	51.7	63.5	59.3
Muscle	72.9	67.5	89.9	80.3
Nerve	5.2	6.3	0	4.3
Parotid	30.9	35.1	49.8	47
Skin	88.3	81.9	98.9	47
Vein	96.6	92.7	7.9	8.3

ROC curves for the different tissue types classified by ARRInet-M and ARRInet-W represent tissue classification accuracy in a different way and reveal the same results (see Supplemental Material). For MSI image classification, the area under the ROC curve (AUC) values ranged from 0.77 for classification of bone and nerve to 1.00 for classification of cartilage, dura, fascia, and skin. For RGB image classification, the AUC values ranged from 0.76 for classification of vein to 1.00 for classification of skin. Differences in AUC values were all determined to be statistically significant (p<0.001).

The tissue classification errors (Table 4) and ROC curves (Supplemental Material) both reveal that ARRInet-M performed better than ARRInet-W for most tissue types but did not perform better than ARRInet-W for some tissue types. These results are consistent with the observation that removing some of the high SNR spectral channels actually decreased classification accuracy for bone, fat, muscle, parotid, and skin. An analysis of tissue confusion matrices provides insight into the decision-making processes of the CNN (see Sec. 4).

**Fig. 5:**
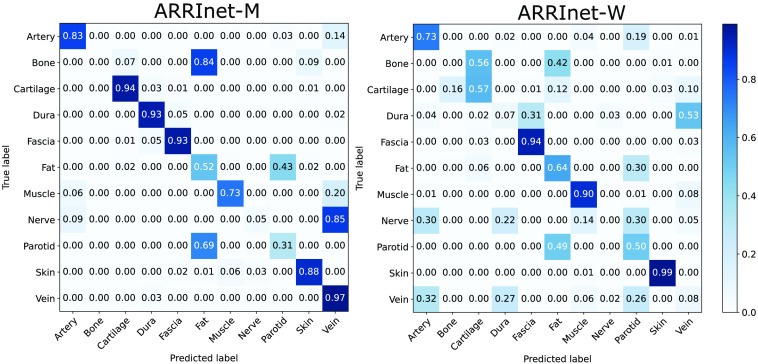
Tissue confusion matrices representing the percentage of tissue types that are correctly and incorrectly classified by the ARRInet-M and ARRInet-W for the test dataset. Confusion matrices for the train and validation datasets are available in the Supplemental Material. The color bar indicates the percentage of tissues with true label (row) that were predicted as another tissue type (column). Diagonal matrix elements are correct predictions and off-diagonal elements are incorrect predictions.

Figure 5 shows a tissue confusion matrix representing the percentage of tissue types that are correctly and incorrectly classified by the ARRInet-M and ARRInet-W. For most tissue types, the percentage of times it was classified correctly (corresponding to the diagonal entries in the tissue confusion matrix and referred to as “true positives”) was greater than the percentage of times it was classified incorrectly (all other entries in the tissue confusion matrix referred to as “true negatives”). However, both ARRInet-M and ARRInet-W failed to classify bone. ARRInet-M classified bone as fat and ARRInet-W classified bone as cartilage or fat. Conversely, both networks did not classify fat as bone. As noted earlier, removing spectral channels did not reduce or increase the classification errors for bone (see Fig. 3). This observation indicates that the MSI system did not provide information that the ARRInet-M could use to classify bone. Without meaningful data about diffuse reflectance, the neural network uses other information, such as the number of times the tissue class is represented, to classify the tissue (see Table 2). There were more samples of fat tissue, and hence the networks selected fat instead bone.

Both ARRInet-M and ARRInet-W failed to classify nerve. ARRInet-M classified nerve as vein and ARRInet-W classified nerve as artery, dura, muscle, and parotid. As in the case for bone, removing spectral channels did not affect classification accuracy (see Fig. 3). And, as in the case for bone, there were more training patches for vein, artery, dura, muscle, and parotid than there were for nerve. Finally, both networks confused parotid and fat. These misclassifications may be in part explained by the fact that fatty tissue is an intrinsic component of the parotid glands.^37^

**Fig. 6:**
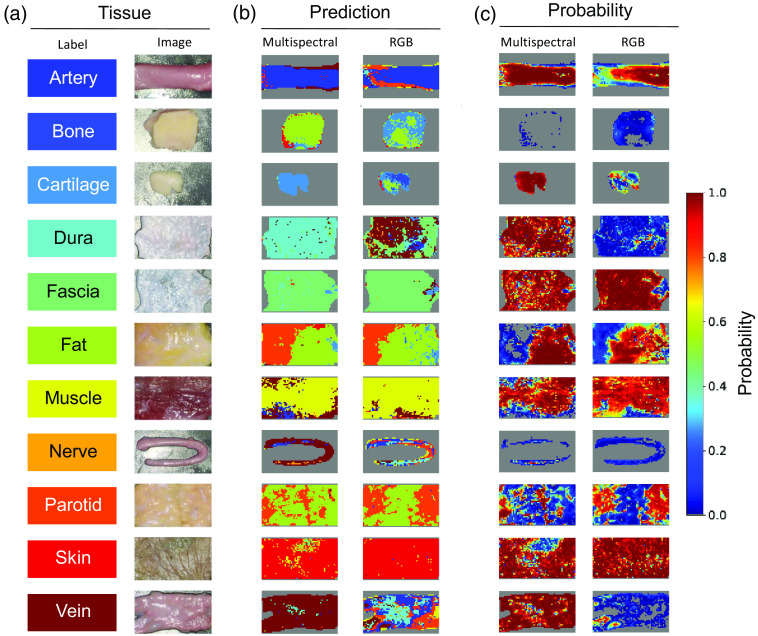
Visualization of correctly and incorrectly classified areas in test images by the multispectral (ARRInet-M) and non-multispectral (ARRInet-W) deep learning models. (a) The test dataset contains one image of each of the 11 tissue types. Broad-band white light illuminated images are shown, and were processed and rendered as TIFF images for illustration purposes only. The raw unprocessed image data (not shown) were used to train the deep-learning neural networks. (b) Label maps of predicted tissue types are shown for all foreground image patches of size 32×32  pixels. Background patches are colored gray and were not classified. The first column of panel (a) shows the color-label key. (c) Probability heatmaps of correct tissue predictions in foreground image patches by the deep learning models.

Figure 6 illustrates the consequences of the tissue misclassifications made by the deep learning models. Areas of correctly and incorrectly identified tissues in test images were visualized using color-coded label maps and probability heatmaps. Each test image was reconstructed from its constituent 32×32  pixel patches that were input to the ARRInet-M and ARRInet-W models, with the patches colored to indicate the predicted tissue type or probability score of a correct tissue prediction at that patch’s location. The prediction label maps shown in Fig. 6(b) and the probability heat maps shown in Fig. 6(c) illustrate the effect of the misclassifications that are quantified by the tissue confusion matrix shown in Fig. 5.

#### 3.3.1 Binary classification of nerve and parotid tissues

**Fig. 7:**
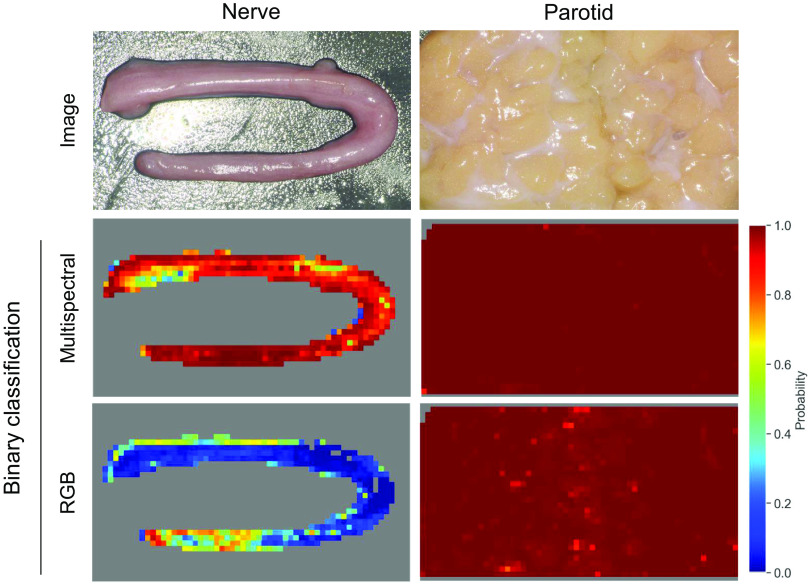
Visualization of correctly and incorrectly classified areas of nerve and parotid test images, for the case of two-class binary classification, by the multispectral (ARRInet-M) and non-multispectral (ARRInet-W) deep learning models. Color-coded heatmaps display the probability of the correct tissue prediction in each 32×32  pixels tile containing tissue foreground.

Clinical context provides important information about which tissues are most likely to be encountered and are most important to identify during surgery. This information can influence tissue predictions made by deep learning classifiers by altering the prior probabilities and weights for assigning predictions. To adapt the models for the clinical context of parotidectomy surgery, we evaluated the specific application of ARRInet-M and ARRInet-W to the task of binary classification of nerve and parotid tissues. Multiclass outputs of the models were binarized by using the output probabilities of parotid and nerve tissue predictions and excluding predictions of other tissue types. Binary classification results for the test set images demonstrate excellent classification of parotid tissue by ARRInet-M and ARRInet-W, with accuracies of 100% by both models, whereas ARRInet-M demonstrated much higher accuracy for classifying nerve tissue compared with ARRInet-W (97.3% versus 19.5%, p<0.001) (Table 5). Visualizations of correctly and incorrectly predicted nerve and parotid image patches, using prediction label maps and probability heat maps, are shown in Fig. 7. ROC curves for binary classification by both models are shown in Supplemental Material.

**Table 5:** Binary classification results by multispectral and nonmultispectral classifiers, ARRInet-M, and ARRInet-W, respectively, for nerve and parotid test images *ex vivo*. Results are described as the percentage of correctly predicted tiles (“prediction”) and the average probability of a correct tissue prediction (“probability”).

	Multispectral imaging		RGB imaging	
Tissue type	Prediction (%)	Probability (%)	Prediction (%)	Probability (%)
Nerve	93.2	87.7	19.5	22.9
Parotid	100	99.9	100	99.9

Binary classification accuracy was higher than multi-class classification accuracy overall in part because of the higher baseline accuracy of random guessing for binary compared with multi-class classification. The accuracy of random guessing is 1/2 and 1/11 for binary and multi-class classification, respectively, not accounting for class imbalances. The higher accuracy of parotid classification compared with nerve classification reflects the class imbalance in the dataset, which had more parotid images than nerve images.

**Fig. 8:**
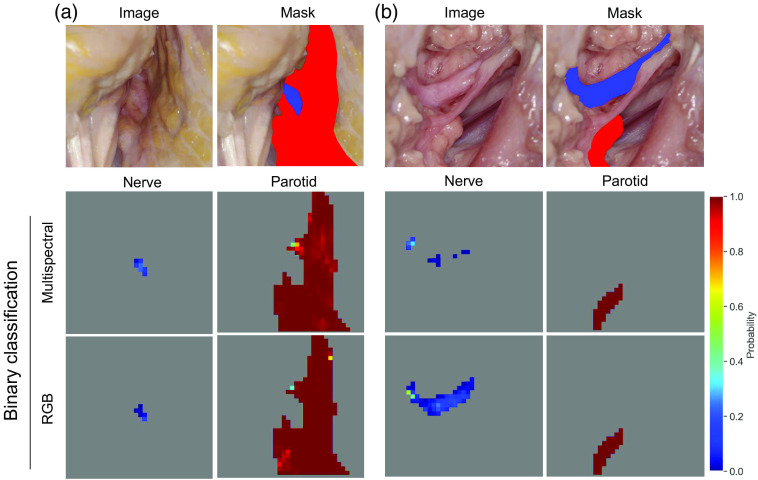
*In situ* images and visualization of nerve and parotid binary classification performance in the in situ images by the deep learning models. Top row: *in situ* images of overlapping (a) or adjacent (b) nerve and parotid tissue. Masks show ground truth segmentations of nerve (blue) and parotid (red) tissues. Middle and bottom rows: color-coded heat maps of the probability of the correct tissue prediction by the multispectral (Arrinet-M) and non-multispectral (Arrinet-W) classifiers.

Visualizations of correctly and incorrectly classified areas in the in situ images are shown in Fig. 8. We evaluated the classification accuracy of ARRInet-M and ARRInet-W when applied to these previously unseen images of parotid and nerve tissues in situ, with the two tissues in overlapping [Fig. 8(a)] or adjacent [Fig. 8(b)] configurations. Evaluation of two in situ images demonstrated excellent classification of parotid tissue with accuracy greater than 99% by both models, but poor classification performance for nerve tissue with accuracies less than 10% by both models (Table 6).

**Table 6:** *In situ* image binary classification results by multispectral and non-multispectral classifiers, ARRInet-M and ARRInet-W, respectively, for nerve and parotid test images. Results are described as the percentage of correctly predicted tiles (“prediction”) and the average probability of a correct tissue prediction (“probability”).

	Multispectral imaging		RGB imaging	
Tissue type	Prediction (%)	Probability (%)	Prediction (%)	Probability (%)
Nerve	0	6.5	1.6	6.2
Parotid	100	99.6	100	99.7

It is important to highlight the fact that ARRInet-W and ARRInet-M were trained on 32×32  pixel patches. This eliminated anatomical features that can help surgeons identify different tissue structures in the operating room. We assume that spectral reflectance was the main source of information available to the neural networks, although it is possible that tissue texture was also a useful source of information.

## 4 Discussion

We created an MSI system by combining a fully digital (RGB) operating microscope with several broad and narrowband illuminants. We captured RGB and MSI images of human head and neck tissues *ex-vivo*, segmented the tissue images, and divided them into nonoverlapping image patches. Deep convolutional neural networks (CNNs) were trained to classify the RGB and MSI image patches by tissue type. On average, tissue classification accuracy was better for networks trained on MSIs than networks trained on RGB images.

We analyzed the performance of the deep convolutional neural network that was trained to classify MSIs of *ex vivo* human tissue and identified two governing principles. First, the network gives more weight to data captured by spectral channels that have high signal-to-noise ratio (SNR). Second, when spectral channels do not provide useful data, the network relies on class priors.

The spectral channel SNR predicted classification accuracy in the following ways. First, there is a high correlation between spectral channels ranked by the occlusion analysis and by SNR. Second, removing imaging data captured by spectral channels with SNR<20  dB had no effect on classification accuracy, indicating that there is a lower bound on channel SNR.

The second principle that governs the performance of deep convolutional neural networks is the fact that in the absence of discriminative sensor data, the network will rely on class priors. The occlusion sensitivity analysis allowed us to identify spectral channels that did not provide useful data for classifying certain tissue types–namely, bone, fat, and parotid. Analysis of tissue confusion matrices show that in the absence of useful sensor data, the network selected the tissue type that was most frequently represented in the training dataset. For example, bone, fat, and parotid were more frequently classified as fat (Fig. 5) because fat was more frequently represented in the training dataset (Table 2).

Understanding the principles governing the CNN classification decisions helps to build trust in their use. We used the occlusion analysis to gain an understanding of how the CNN classified different tissue types. When available, the CNN used information from spectral channels with high SNR to classify different tissue types. In the absence of this information, the CNN used class priors to classify different tissue types. In addition to understanding CNN decision-making, we wish to experiment with new designs for MSI systems. Toward this end, the spectral channel SNR can be used to evaluate the effectiveness of each spectral channel in a MSI system.

Spectral channel SNR is a useful and perhaps necessary performance metric, but it is clearly not sufficient. The properties of tissue reflectance is a fundamental constraint on the ability of any imaging system to differentiate between different tissue types. In this study, we were limited by the spectral power of the illuminants that were available and the spectral sensitivities of the RGB imaging sensor in the ARRIScope digital microscope. In the future, we plan to customize the design of spectral channels based on measurements of tissue reflectance properties.

RGB imaging sensors undersample tissue reflectance. Hyperspectral imaging sensors oversample tissue reflectance. The goal of designing a MSI system for surgical applications is to reduce the number of spectral channels that are necessary for classifying tissue types and/or delineating tumor margins. By modeling both tissue reflectance and MSI spectral channels, we can both test our understanding and optimize the design of MSI systems for classifying different tissue types.

In the future, there will be many more design options for creating MSI systems. Advances in the manufacturing of thin-film filters make it possible to customize the design of multispectral color filter arrays. Looking farther ahead, we can envision the impact that advances in optical metasurfaces will have on the design of MSI systems. Our results show that the spectral channel SNR will be a useful metric for determining the effectiveness of new types of MSI systems for surgical applications.

## 5 Conclusion

Tissue classification accuracy was slightly better for networks trained on MSIs than networks that were trained on RGB images, supporting the conclusion that increasing the number of spectral channels provides additional information that can be used to distinguish different tissue types. Occlusion analysis revealed that the network used information from spectral channels with high SNR to classify different tissue types. In the absence of this information, the network used class priors to classify different tissue types. The impact that each additional spectral channel had on tissue classification can be quantified by the channel SNR.

## Code, Data, and Materials Availability

Data underlying the results presented in this paper are not publicly available at this time but may be obtained from the authors upon reasonable request. See Supplemental Material for supporting content.
