# Supplementary material for: Signal to noise ratio quantifies the contribution of spectral channels to classification of human head and neck tissues ex vivo using deep learning and multispectral imaging
Source: J Biomed Opt. 2023 Jan 28;28(1):016004. doi: 10.1117/1.JBO.28.1.016004 (PMC9884103; doi:10.1117/1.JBO.28.1.016004)
Supplement: Supplementary file 1 [file JBO_028_016004_SD001.pdf]

Signal to noise ratio quantifies the contribution of spectral channels to classification of human head and neck tissues *ex vivo* using deep learning and multispectral imaging: supplemental materials

George S. Liu[a], Jared A. Shenson[a], Joyce E. Farrell[b], Nikolas H. Blevins[a,*]
[a]Stanford University, Department of Otolaryngology–Head and Neck Surgery, 801 Welch Road, Palo Alto, California, USA, 94304
[b]Stanford University, Department of Electrical Engineering, 350 Jane Stanford Way, Stanford, California, USA, 94305

Keywords: multispectral imaging, machine learning, classification, lasers, sensor, tissue.

*Nikolas H. Blevins, nblevins@stanford.edu

1 Estimation of the ARRIscope spectral sensitivity

The digital value for each pixel in a digital camera is determined by many factors, most notably the number of photons incident on the sensor array, the quantum efficiency of the sensor array, pixel size, fill factor, conversion gain and exposure duration. The number of photons incident on the sensor array is determined by the spectral energy in the incident light, the spectral transmittance of optical components (including lens, microlens and filters placed between the light and sensor. Other factors that modulate the digital values of each pixel are optical and electrical crosstalk and electronic gain.

The combined effect of the spectral transmittance of the optical elements (including lens and filters), the quantum efficiency of the imaging sensor, crosstalk and gain can be represented by spectral sensitivity functions for the R, G and B sensors. It is possible to directly measure the spectral sensitivities of a CMOS imaging sensor by recording pixel R, G and B sensor values for narrowband spectral lights spanning the range between 400 and 950 nm. Since we were not able to make these measurements, we developed a method for estimating the spectral sensitivities of the

R, G and B sensors based on pixel values obtained from ARRIScope images of a color calibration target illuminated with 6 different lights.

1.1 Estimation method

We captured raw (unprocessed) ARRIScope image data of a color calibration chart as it was illuminated (sequentially) with 6 different spectral lights. In separate experiments, we confirmed that the RGB pixel values in the raw images increased linearly with light intensity.

We measured the spectral reflectances of each of the 24 color patches in the color calibration chart (a miniature version of the Macbeth Color Checker), hereafter referred to as the MCC. We also measured the spectral radiance of each of the 6 different spectral lights. Spectral measurements were made using a PR-715 SpectraScan spectroradiometer (Photo Research).

The six lights included the broadband light source of the ARRIScope, the broadband light source of the Sony illumination system, and 4 of the 5 Sony narrowband lights sources (with peak energy at 405, 445, 525 and 638 nm). Since we estimated the spectral sensitivities of the ARRIScope with the NIR blocking filter in the "on" position, we did not include the Sony narrowband light with peak wavelength at 808 nm.

Figure 1A plots the spectral reflectances of a miniature version of the MCC target that was placed within the field of view of the ARRIScope sensor. Figure 1B plots the spectral energy in each of the 6 lights that were used for this calibration on a log10 scale in order to illustrate that the spectral energy of the broadband light in the ARRIscope is several orders of magnitude higher than the spectral energy in the Sony broadband and narrowband lights. We captured images of the color calibration target illuminated with the 6 different lights at the same exposure duration. Consequently, the SNR of the image data is determined by the spectral energy in the light and the

spectral sensitivities of the R, G and B sensor in the ARRIscope.

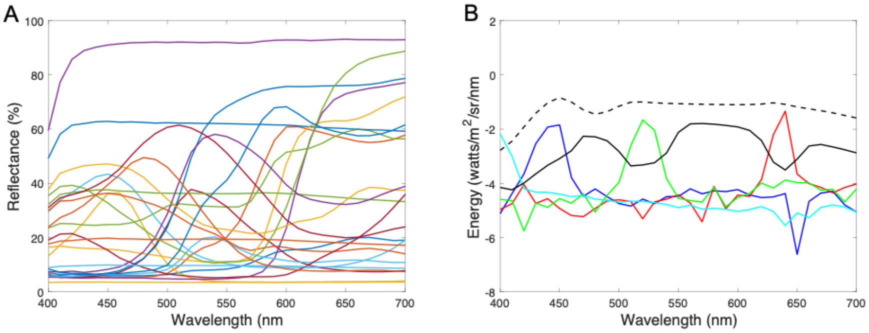

Fig 1 Surfaces and lights used in sensor estimation method. A) The left figure plots the spectral reflectance of the 24 color patches in the MCC color calibration target. B) The right figure plots the spectral energy in each of the 5 lights in the Sony illumination device and the ARRIScope broadband light. The spectral energy is plotted on a log10 scale.

We estimate the spectral sensitivities of the RGB sensors + NIR blocking filter in the following way. First, we represent the spectral radiance of each of the 24 color patches under each of the 6 lights (144 spectral stimuli) by a Wx144 matrix, E, where W is the number of wavelength samples. (For example, if we sample every 10 nm between 400 and 700 nm, then W would be 31).

We model the sensor spectral sensitivities of each channel as the weighted sum of 7 Gaussians, 30 nm bandwidth, centered at wavelengths ranging between 400 and 700 nm, in steps of 50 nm. Suppose the basis functions are in the columns of the matrix G (Wx7), and the weights for each sensor is in the columns of the matrix S (7x3). In this case GS (Wx3) contains the sensor spectral sensitivities in the columns.

The RGB camera responses, R, to the inputs in the columns of E should be

$$R = (GS)^t E = S^t G^t E \tag{1}$$

For simplicity, suppose $G^t E = B$. Then,

$$R = S^t B \tag{2}$$

$$S^t = R(B)^t(BB^t)^{-1} \tag{3}$$

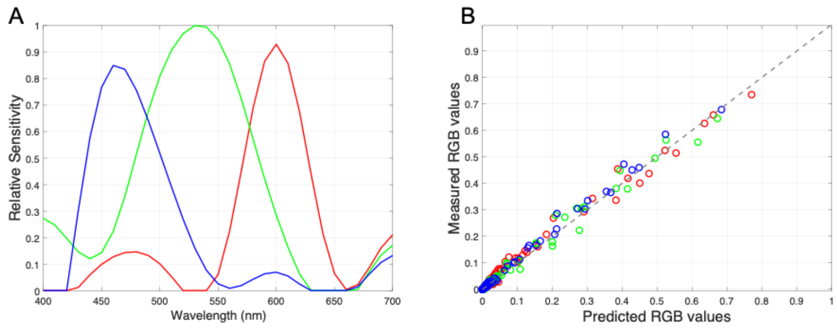

Fig 2 Estimated Spectral Sensitivity Functions. A) Estimated spectral sensitivities for the ARRIScope RGB sensors. B) Measured RGB values for the 24 color patches illuminated by 6 different lights plotted against the RGB values predicted by the estimated sensors.

In this experiment R is a 3x144 matrix containing the mean camera RGB values for 24 color patches illuminated by 6 different lights. Everything in the equation is known except for the weights, S. We estimate S using least-squares methods (pseudo-inverse) and compute GS to be the estimated spectral sensitivities of the ARRIScope RGB sensors. Figure 2A plots the estimated spectral sensitivities (GS) and Figure 2B plots the measured RGB values for the 24 color patches in the MCC against the predicted RGB values ($(GS)^t E$).

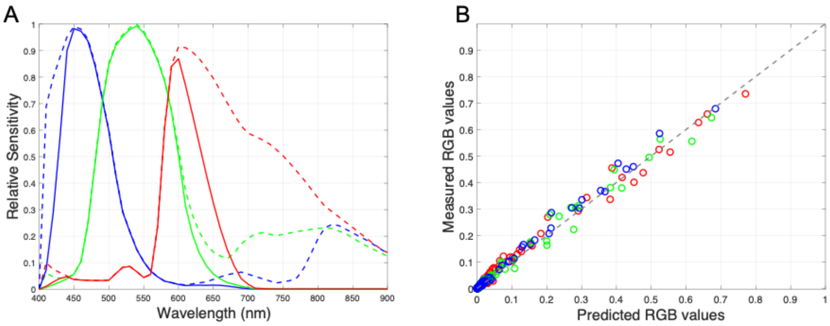

Fig 3 Equivalent Spectral Sensitivity Functions. A) Spectral sensitivities of an unnamed sensor with (solid line) and without (dashed line) an UV + NIR blocking filter. B) Mean RGB values for the 24 color patches illuminated by 6 different lights predicted by the spectral sensitivities for the unnamed sensor with the UV + NIR blocking filter.

1.2 Equivalent sensor spectral sensitivities

The estimated spectral sensitivity functions shown in Figure 2A are not the only functions that can predict the measured RGB values for the 24 color patches illuminated by the 6 illuminants. This is because our method for estimating the sensor spectral sensitivities is limited by the low-dimensionality of the spectral reflectances of surfaces in the MCC and the lights. These surfaces and lights do not adequately sample the wavelengths that the R, G and B pixels can capture.

The measured RGB values for the 24 color patches illuminated by the 6 different illuminants can also be predicted by the spectral sensitivity functions published in the EBU (European Broadcast Union) 2012 standard for the "Television Lighting Consistency Index".[38] We also compared the estimated spectral sensitivity functions to spectral sensitivity functions for a different RGB ARRIScope published by Wisotzky et al. (2019)[39] and found that the differences can be explained by an adjustment in gain and in the properties of UV and NIR blocking filters.

The estimated and published spectral sensitivity functions for the ARRIScope RGB sensors include the effect of an NIR blocking filter. However, there was one condition in which we captured

images of tissue samples illuminated with an 808 nm light and the NIR blocking filter in the "off" position. In order to calculate the SNR of the R, G and B sensors in this condition, we need to remove the effect of the NIR blocking filter. Figure 3 plots an equivalent sensor model with and without a broadband filter that blocks UV and IR. We do not know if there was a separate UV blocking filter in place. It is more likely that UV was blocked by other optical components in the ARRIScope imaging system. CMOS imaging sensors also have poor sensitivity in the UV range.

2 Maximum permissible exposure of lights

Although it is not necessary for researchers and clinicians to view the illuminated tissue, all of the lights we used had radiant powers that were well below the MPE limits defined by IEC 62471:2006 and ANSI X136.1:2014 . Hence, the lights do not pose a hazard to either the illuminated tissue or to a human retina. The software that we used to calculate the MPE limits for different applications, as well as sensor SNR, can be found in https://github.com/ISET/isetcam.

3 Supplemental Figures

Figure 4 shows example TIFF images obtained by the MSI system for each of the 11 tissue types.

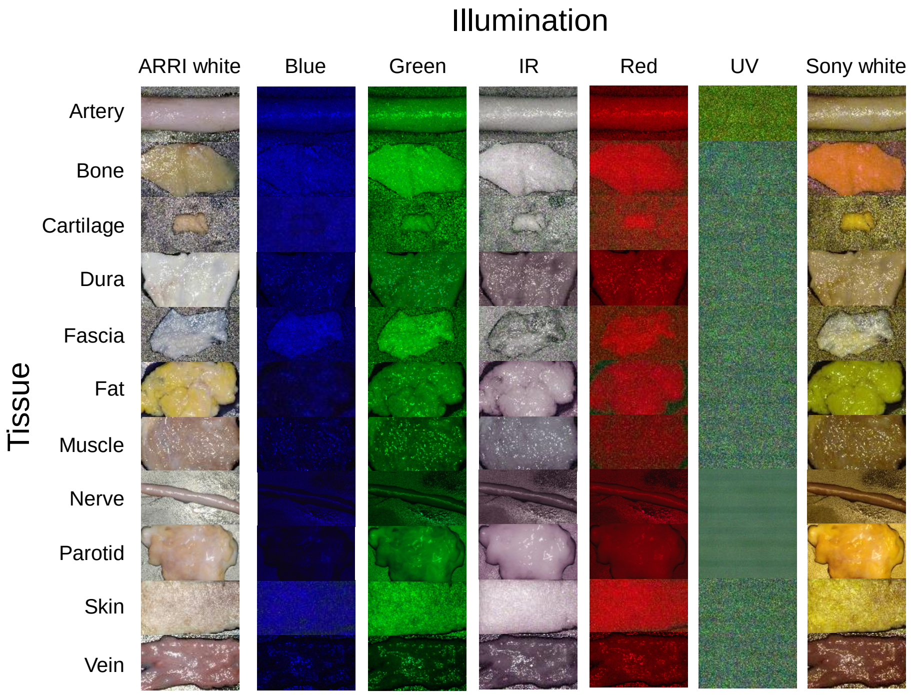

Fig 4 TIFF images of 11 of the 92 tissue specimens that were excised and imaged by the MSI system. Images were acquired ex-vivo using sequential illumination with broad-band white, blue, green, infrared (IR), red, ultraviolet (UV), and narrow-band white light. An RGB image, representing the output of 3 spectral channels, was captured for each illumination condition. UV images lacked apparent visual information and were omitted from later analysis. The remaining 6 lights and 3 RGB sensors.

Figure 5 shows receiver operating characteristic (ROC) curves for different tissue types classified by ARRInet-M and ARRInet-W.

Figure 6 shows ROC curves for binary classification of nerve and parotid tissues by ARRInet-M and ARRInet-W models.

Figure 7 shows confusion matrices for the classification of train and validation images by the ARRInet-M and ARRInet-W models.

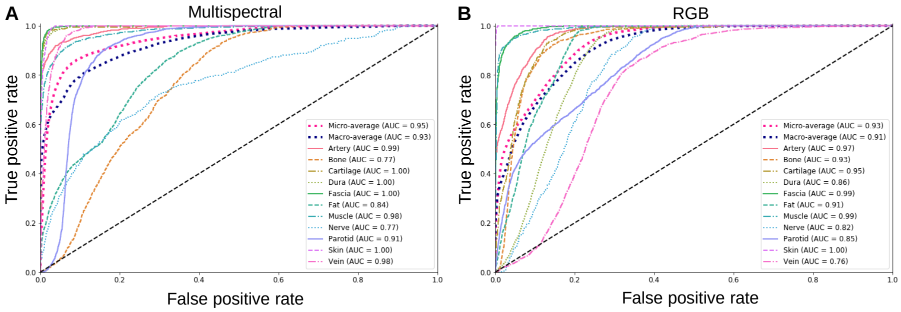

Fig 5 Receiver operating characteristic (ROC) curves for classification performance on test image tiles by the multispectral (ARRInet-M, left) and non-multispectral (ARRInet-W, right) deep learning models. ROC curves were calculated using one-versus-all, micro average, and macro average measures of multiclass classification performance. The dashed, black line indicates the ROC curve for random guessing.

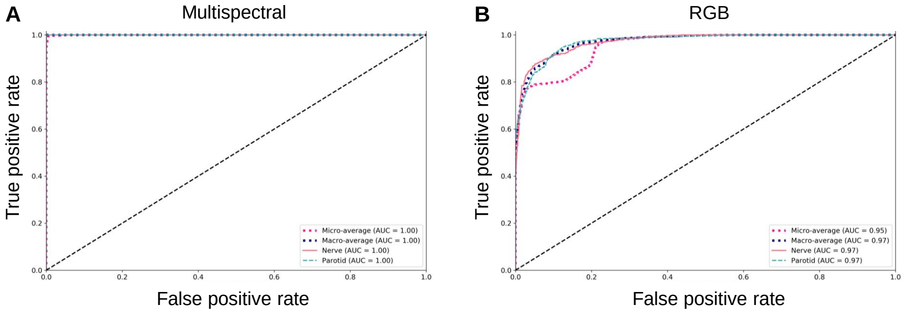

Fig 6 Receiver operating characteristic (ROC) curves for binary classification performance by the multispectral (ARRInet-M, left) and non-multispectral (ARRInet-W, right) deep learning models to distinguish nerve and parotid test image tiles. The dashed, black line indicates the ROC curve for random guessing.

List of Figures

1 Surfaces and lights used in sensor estimation method. A) The left figure plots the spectral reflectance of the 24 color patches in the MCC color calibration target. B) The right figure plots the spectral energy in each of the 5 lights in the Sony illumination device and the ARRIScope broadband light. The spectral energy is plotted on a log10 scale.

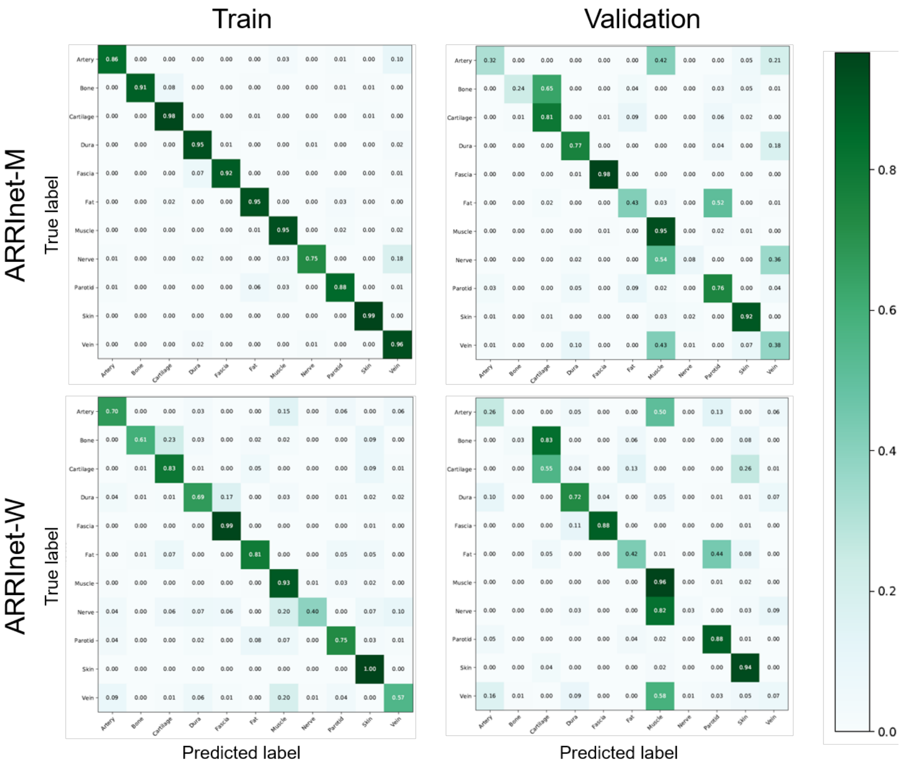

Fig 7 Tissue confusion matrices representing the percentage of tissue types that are correctly and incorrectly classified by the ARRInet-M and ARRInet-W for the train and validation datasets.

2 Estimated Spectral Sensitivity Functions. A) Estimated spectral sensitivities for the ARRIScope RGB sensors. B) Measured RGB values for the 24 color patches illuminated by 6 different lights plotted against the RGB values predicted by the estimated sensors.

3 Equivalent Spectral Sensitivity Functions. A) Spectral sensitivities of an unnamed sensor with (solid line) and without (dashed line) an UV + NIR blocking filter. B) Mean RGB values for the 24 color patches illuminated by 6 different lights predicted by the spectral sensitivities for the unnamed sensor with the UV + NIR blocking filter.

4 TIFF images of 11 of the 92 tissue specimens that were excised and imaged by the MSI system. Images were acquired ex-vivo using sequential illumination with broad-band white, blue, green, infrared (IR), red, ultraviolet (UV), and narrow-band white light. An RGB image, representing the output of 3 spectral channels, was captured for each illumination condition. UV images lacked apparent visual information and were omitted from later analysis. The remaining 6 lights and 3 RGB sensors.

5 Receiver operating characteristic (ROC) curves for classification performance on test image tiles by the multispectral (ARRInet-M, left) and non-multispectral (ARRInet-W, right) deep learning models. ROC curves were calculated using one-versus-all, micro average, and macro average measures of multiclass classification performance. The dashed, black line indicates the ROC curve for random guessing.

6 Receiver operating characteristic (ROC) curves for binary classification performance by the multispectral (ARRInet-M, left) and non-multispectral (ARRInet-W, right) deep learning models to distinguish nerve and parotid test image tiles. The dashed, black line indicates the ROC curve for random guessing.

7 Tissue confusion matrices representing the percentage of tissue types that are correctly and incorrectly classified by the ARRInet-M and ARRInet-W for the train and validation datasets.
